# Omeprazole Treatment Enhances Nitrogen Use Efficiency Through Increased Nitrogen Uptake and Assimilation in Corn

**DOI:** 10.3389/fpls.2019.01507

**Published:** 2019-12-04

**Authors:** Michael James Van Oosten, Emilia Dell’Aversana, Alessandra Ruggiero, Valerio Cirillo, Yves Gibon, Pasqualina Woodrow, Albino Maggio, Petronia Carillo

**Affiliations:** ^1^Department of Agricultural Sciences, University of Naples Federico II, Portici (NA), Italy; ^2^Department of Environmental, Biological and Pharmaceutical Sciences and Technologies of University of Campania “Luigi Vanvitelli”, Caserta, Italy; ^3^UMR 1332 BFP, INRA, Bordeaux INP, Villenave d’Ornon, France

**Keywords:** benzimidazole, nitrogen use efficiency, proton pump inhibitor, maize, nitrogen assimilation, nitrate reductase

## Abstract

Omeprazole is a selective proton pump inhibitor in humans that inhibits the H^+^/K^+^-ATPase of gastric parietal cells. Omeprazole has been recently shown to act as a plant growth regulator and enhancer of salt stress tolerance. Here, we report that omeprazole treatment in hydroponically grown maize improves nitrogen uptake and assimilation. The presence of micromolar concentrations of omeprazole in the nutrient solution alleviates the chlorosis and growth inhibition induced by low nitrogen availability. Nitrate uptake and assimilation is enhanced in omeprazole treated plants through changes in nitrate reductase activity, primary metabolism, and gene expression. Omeprazole enhances nitrate assimilation through an interaction with nitrate reductase, altering its activation state and affinity for nitrate as a substrate. Omeprazole and its targets represent a novel method for enhancing nitrogen use efficiency in plants.

## Highlight

Omeprazole enhances nitrogen uptake and assimilation in corn.

## Introduction

In recent decades, increasing nitrogen use efficiency (NUE) has emerged as critical target for reducing the requirements for chemical fertilizers and lessen the potential environmental impacts of nitrogen fertilization in agriculture. The Green Revolution resulted in dramatic increases in crop productivity allowing modern agriculture to keep up with a tripling in the global population over the last seventy years (Godfray et al., 2010). However, higher yield and productivity were in part achieved through a sevenfold increase in nitrogen fertilizers use (Hirel et al., 2007; Lassaletta et al., 2014), which, in turn, had critical consequences on the environment. The chemical synthesis of ammonia, ammonium, urea, and nitrate for use in agriculture through the Haber-Bosch process produces 450 million tons of chemical fertilizer per year consuming up to 5% of the global natural gas production (Smith, 2002; Smill, 2004; Erisman et al., 2008; Chen et al., 2018). It is estimated that 50% of the nitrogen atoms in humans today originate from nitrogen fixed from the Haber-Bosch process (Erisman et al., 2008). Moreover, for each metric ton of NH_3_ produced 1.9 metric tons of CO_2_ are released into the environment (Rafiqul et al., 2005). Overuse and poor management of chemical fertilizers, in particular in developing countries, have caused environmental degradation and pollution and reduction of the arable land (Keeney and Hatfield, 2001). Among fertilizers, nitrogen (N) represents the single highest input cost for many crops and, since its production is energy intensive, this cost is dependent on the price of energy (Rothstein, 2007). N containing compounds, which are typically used as chemical fertilizers, are generally mobile in the soil, and only 30%– 40% of the applied N is taken up by plants (Raun and Johnson, 1999), with the remainder lost for leaching, surface run-off, denitrification, volatilization, and microbial consumption. Increasing NUE by only 1% could globally save $2.3 billion annually (Raun and Johnson, 1999). Based on indications by Hirel et al. (2007), the Organization for Economic Co-operation and Development (OECD) described NUE as the ratio between the amount of N fertilizer exported from the field by the crops and the amount of N fertilizer applied. However, NUE depends not only on the plants’ ability to uptake nitrate but also on nitrate assimilatory reduction and translocation efficiency (Reich et al., 2014). Until now, crops, like maize, have been selected almost exclusively under nonlimiting N conditions. In contrast, attempts to reduce the use of nitrogen fertilizers have faced the inability of crop plants to adapt to a low nitrogen availability leading to significant decrease in crop yield (Masclaux-Daubresse et al., 2010). Nitrogen is of pivotal importance in plant metabolism. Nitrate reductase (NR), the first enzyme in the nitrate assimilation pathway catalyzing the reduction of NO_3_^-^ in NO_2_^-^ is strictly dependent on nitrate availability at cellular level (Carillo et al., 2005; Annunziata et al., 2017). This enzyme is considered as the limiting step in the overall process of plant growth and productivity (Kaiser et al., 1999). Nitrate is required for full levels of NR gene expression, since signals from nitrogen metabolism play an important role in inducing the expression of NR gene Nia (Oaks, 1974).

It is therefore critical to better understand the physiological and molecular basis of N assimilation and use in plants to design new strategies to improve NUE.

Omeprazole (OP) is a member of the family of substituted benzimidazoles that act as proton pump inhibitors (PPIs) in mammals (Baumann and Baxendale, 2013). OP interacts with P-Type IIC ATPases and inhibit H^+^/K^+^ ATPases in the gut lumen, reducing the pacification of the gut (Shin et al., 2009). Plants do not appear to have Type IIC ATPases that move potassium or sodium across the membrane; instead they transport Na^+^ and K^+^ using the family of NHX-type antiporters (Axelsen and Palmgren, 1998; Hasegawa, 2013). Notwithstanding plants do not possess functional orthologues of H^+^/K^+^ ATPases, OP does influence the physiological processes of the plant (Sweadner and Donnet, 2001). OP has previously been shown to act at micromolar concentrations in tomato and basil as an enhancer of growth (Van Oosten et al., 2017a; Carillo et al., 2019d; Cirillo et al., 2019) and it was proven to enhance nitrogen and potassium uptake and their loading into the shoots in lettuce and basil (Carillo et al., 2019c; Carillo et al., 2019d). OP also increased plant tolerance to salt stress through several adaptive mechanisms (Van Oosten et al., 2017a; Van Oosten et al., 2017b; Rouphael et al., 2018; Carillo et al., 2019c; Carillo et al., 2019d). While the targets of OP that are responsible for these responses in terms of growth and stress tolerance are unclear, it was quite evident that OP affects plant metabolism and NUE on many levels. We hypothesized that OP could interact directly with mechanisms of nitrate uptake and assimilation and that a study examining the role of OP on plants under nitrogen stress would be informative. Therefore, we determined if plant metabolism under high (10 mM NO_3_^-^) or low (1 mM NO_3_^-^) inorganic N availability may change in response to OP treatment in order to understand the role played by OP in improving NUE. Here, we demonstrate that addition of micromolar concentrations of OP to the nutrient solution alleviates chlorosis and growth inhibition, induced by low nitrogen availability, through changes in NR activity, primary metabolism, and gene expression.

## Materials and Methods

### Plant Material and Growth Conditions

The p1619 line (Pioneer Hi-Bred International, Johnston Iowa USA) of maize (*Zea mays* L.) was used for the experiments. For hydroponic experiments, maize seeds were imbibed for 48 h in tap water with aeration and germinated on filter paper wetted for three days and transferred to hydroponics. Two modified Hoagland’s solutions supplemented with Hidromix S micronutrients (Vlagro, Cieti Italy) (1g/L) were used: low nitrogen with 1mM NO_3_^-^ in test-run experiments showed clear signs of nitrogen stress with reduced growth and chlorosis while high nitrogen with 10mM NO_3_^-^ demonstrated excellent growth and no signs of nitrogen stress. Therefore, the selected concentrations (1 vs. 10 mM NO_3_^-^) allowed us to visually differentiate plants growing under optimal vs. suboptimal N availability without causing irreversible metabolic dysfunctions and cell death in the short term (Carillo et al., 2008). Three replicates containing six plants each were made for each nutrient regimen and OP treatment. The OP at final concentration of 1 µM was supplied to the nutrient solution to a set of replicates for OP treatment starting from 14 days after germination. The 1 µM OP was selected based on previous experiments in which this concentration was found optimal as growth enhancer (Van Oosten et al., 2017; Cirillo et al., 2019). Nutrient solutions with and without OP were changed every four days for the first 2 weeks and every 3 days for the final week of the experiment. Plants were grown in a climate-controlled greenhouse with 8 h of supplemental lighting (1,000 µmol/m^2^/s) and day/night temperature of 28°C/18°C as per Eddy and Hahn (2010).

### Biometric Measurements

At the end of the experiment, 4 weeks DAST, SPAD values (Chlorophyll Meter SPAD-502Plus, Konica Minolta) were measured from 20 leaves of each treatment. Roots and shoots were separated and weighed for fresh weight and total leaf area was calculated using ImageJ (Abramoff et al., 2004). Roots and shoots were then dried for five days at 65°C and dry weight was measured.

### Net Uptake Assay and Kinetic Parameters

The net nitrate uptake rate (NNUR) was measured by a depletion method adapted from (Sorgonà et al., 2011). Maize seeds were imbibed for 48 h in tap water with aeration and germinated on filter paper wetted with one quarter strength Hoagland’s solution with or without 1 µM OP and then transferred to 10 cm × 50 cm trays with washed sand. Sand was kept moist with watering and quarter strength Hoagland’s solution with or without 1 µM OP. Three-week-old maize plants were washed three times and divided into 1-g pools and incubated in 10 ml of apoplastic equilibration solution containing 100 µM KH_2_PO_4_, 250 µM K_2_SO_4_, and 200 µM MgSO_4_. Net nitrate uptake was measured for 1 h and for four biological replicates using 0, 100, and 500 µM KNO_3_^-^ and 0, 1, 10, 50, and 100 µM OP.

### Microsomal Membrane Isolation and ATPase Assays

Total microsomal membranes were isolated as per Yang and Murphy, 2003, using 5 g of separated root and shoot tissue. ATPase activity was measured with an ATPase/GTPase Activity Assay Kit (Sigma-Aldrich, Cat. No. MAK113). Four biological replicates of freshly prepared microsomes from roots and shoots were tested using 10 µl of the microsomal fraction in conjunction with 0, 0.0001, 0.001, 0.01, 0.1, 1, 10, 50, 100, and 1,000 µM omeprazole. Sodium ortho-vanadate (1 mM), a strong suppressor of ATPase activity was added to the negative controls. ATPase activity was measured after a 30-min incubation time at 620 nm.

### RNA Extraction and Quantitative RT-PCR

Roots and shoots were separated and snap frozen in liquid nitrogen at 4 weeks DAST. Total RNA and quantitative RT-PCR was performed as in Van Oosten et al. (2017a). Relative expression levels were calculated using molybdenum cofactor biosynthesis (GRMZM2G067176) as an internal standard (Best et al., 2016). All primers were designed to amplify a cDNA fragment of 120 bp (+/− 10 bp) with an annealing temperature of 55°C (+/− 1°C). All primers were determined to be within 5% efficiency. The ΔΔCt method was used for relative quantification. Three biological replicates were used to calculate the relative expression of each gene. Results were statistically analyzed using Student’s T-Test for each treatment compared to high N controls. Primers used in this study are listed in **Supplementary Table 1**.

### NR Assays in Maize Shoots

NR protein was extracted according to Scheible et al. (1997) from maize. NR activity was assayed by a method modified by Gibon et al. (2004). Leaf extracts (20 µl), as well as nitrite standards ranging from 0 to 10 nmol, were pipetted into microplate wells and incubated with 45 µl medium containing 50 mM Hepes/KOH pH 7.5, 1 mM sodium fluoride, 5 µM Na_2_MoO_4_, 10 µM flavin adenine dinucleotide, 0.5 mM DTT, 15 µM leupeptin, 10 mM potassium nitrate, and 5 mM EDTA (for maximal activity) or 10 mM MgCl_2_ (for selective activity). The reaction was started by the addition of NADH to a final concentration of 0.8 mM. The reaction was stopped with 5 µl of 0.6 M zinc acetate. Then, 15 µl of 0.25 mM phenazine methosulfate (PMS) were added, and the microplates were incubated for 15 min at room temperature. Finally, 120 µl of 1% (w/v) sulphanilamide and 0.02% (w/v) N(1-naphtyl)ethylenediamine dihydrochloride in 3 N HCl were added. After 20 min, the OD was red at 540 nm.

### OP-Dependent Modulation of NR Catalytic Properties

For testing the effects of OP on NR *in vitro*, velocities were determined spectrophotometrically using purified *Arabidopsis thaliana* NR (≥ 0.5 U mg^-1^ protein) obtained from Sigma-Aldrich. Enzyme activity was determined using the assay system described above in presence of OP (0, 1, 10, or 50 µM) in the assay solutions containing nitrate 0.08, 0.16, 0.4, 0.8, 1.5 mM. Global fitting analysis, maximum rate (V_max_) and Michaelis constant (K_m_) were calculated by nonlinear regression using the enzyme kinetics module (SigmaPlot 12.5; Systat Software GmbH, Erkrath, Germany).

### Measurements of Other Enzymes of Central C and N Metabolism

Frozen leaf and root tissues were reduced to a homogenous powder and stored at −80°C until required for the enzyme assays. The extraction buffer consisted of 500 mM HEPES, pH 7.5, containing 100 mM MgCl_2_, 10 mM EDTA, 10 mM EGTA, 2 mM leupeptin, 0.5 mM DTT, 0.1% (v/v) Triton, and 1% (w/v) polyvinylpolypyrrolidone. All extractions were performed at 4°C. A robot-based platform was used to measure the activity of enzymes involved in central C, and N metabolism. ADP glucose pyrophosphorylase (AGPase), glucokinase (GlcK), glucose-6P dehydrogenase (G6PDH), glutamate dehydrogenase (GLDH), phosphoenolpyruvate carboxylase (PEPC), and pyruvate kinase (PK) activities were assayed by using the protocols described by Gibon et al. (2004). Additional methods were used for the cytosolic and mitochondrial citrate synthase (C-CS and M-CS) (Nunes-Nesi et al., 2007), and NADP-, and NAD-isocitrate dehydrogenase (NADP- and NAD-ICDH) (Zhang et al., 2010).

### Quantification of Ions and Metabolites

Ions and organic acids were assayed according to Ferchichi et al. (2018). Primary amino acids and proline were extracted and assayed according to Woodrow et al. (2017). Hydrogen peroxide (H_2_O_2_) amounts were evaluated according to (Baptista et al., 2007). Total proteins, starch, and soluble sugars were determined according to Carillo et al. (2012).

### Statistical Analysis

Shoots from six and twenty plants for each treatment were used for biometric and SPAD measurements, respectively. The other analyses were performed on three biological replicates for each treatment. Biometric measurements were statistically analyzed using the Student’s t-test. The PCA analysis was assess using Minitab 16.8 statistical software (Ciarmiello et al., 2015). The score plot and loading matrix were determined based on the first and second principal components (PCs). A heatmap was generated using the https://biit.cs.ut.ee/CLUSTVIS/online program package with Euclidean distance as the similarity measure and hierarchical clustering with complete linkage. Morpho-physiological parameters and mineral composition data were visualized using a false color scale, with red indicating an increase and blue a decrease of values (Carillo et al., 2019a).

## Results

### Plant Growth

**Figure 1:**
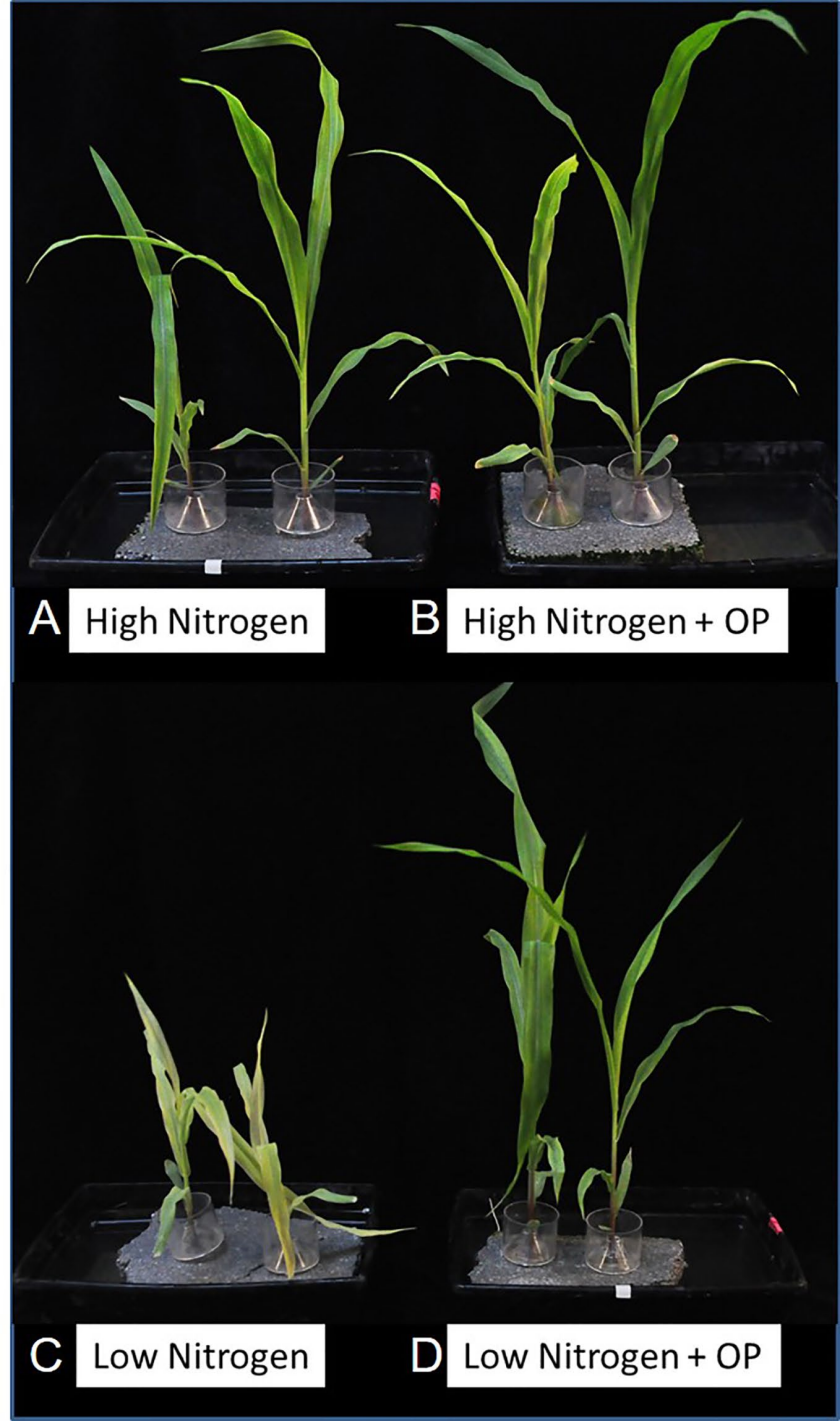
Phenotype of hydroponically grown maize (*Zea mays* L.) plants in nitrogen stress conditions with 1 µM omeprazole (OP). **(A)** Control plants supplied with 10 mM NO_3_^-^ (high N), **(B)** 1 µM OP treated high N plants, **(C)** Nitrogen stressed plants supplied with 1 mM NO_3_^-^ (low N), **(D)** 1µM OP treated low N plants.

**Figure 2:**
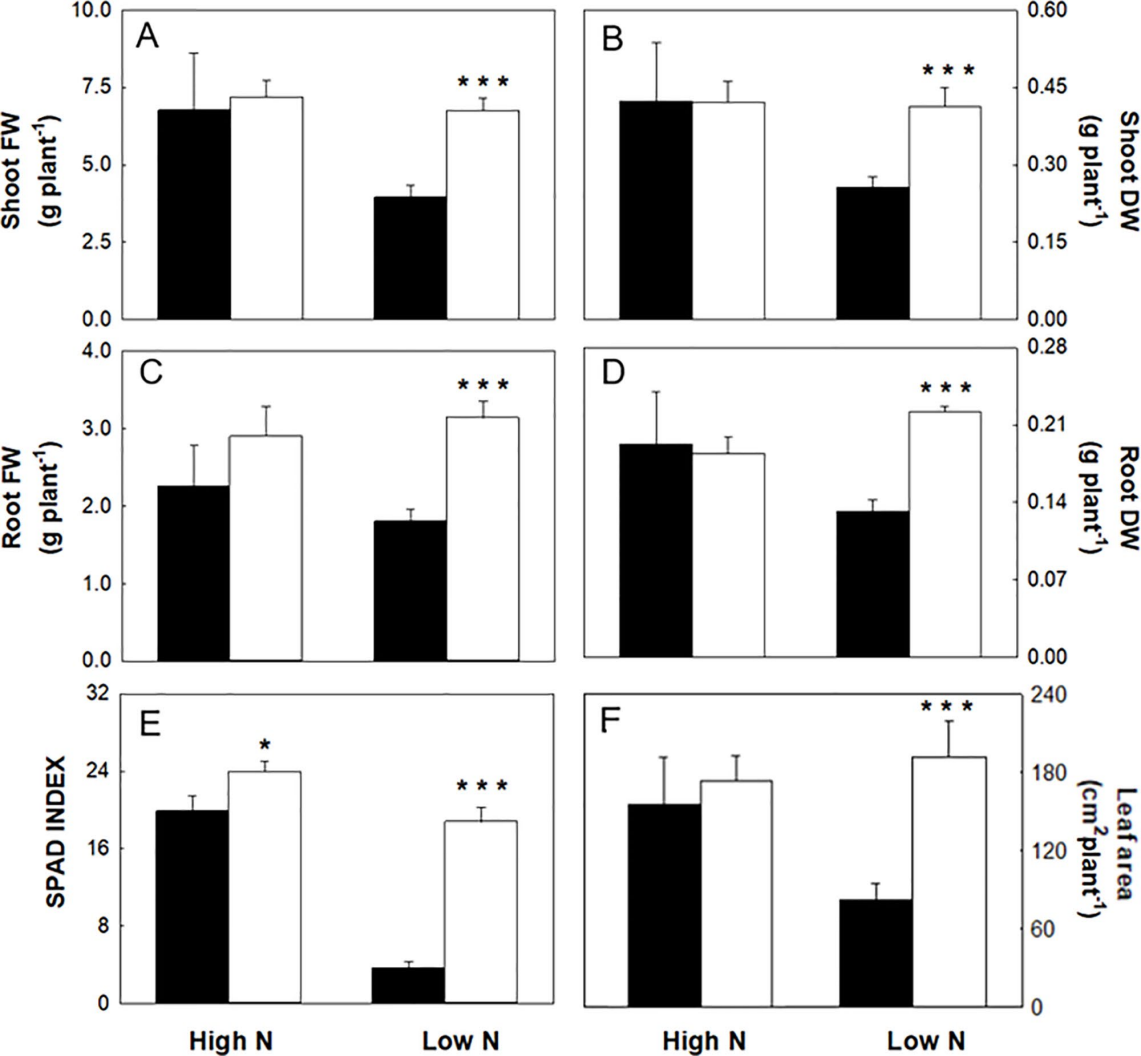
Biometrics and SPAD index of maize plants hydroponically grown in control (10 mM NO_3_^-^, high N) and stress (1 mM NO_3_^-^, low N) nitrogen conditions with (black bars) and without 1 µM omeprazole (OP) (white bars). **(A)** FW of shoots, **(B)** DW of shoots, **(C)** FW of roots, **(D)** DW of roots, **(E)** SPAD index, and **(F)** leaf area. Data are means ± s.d.; n = 6 and 20 plants for each treatment for biometric and SPAD measurements, respectively. Asterisks denote significant differences (*, P < 0.05; ***, P < 0.001) between untreated controls and OP treated plants.

Plants grown in hydroponics with 1 (low N) or 10 mM NO_3_^-^ (high N) with and without 1 µM omeprazole (OP) are shown in **Figure 1**. In nonstress conditions (high N), OP treatment did not significantly increase growth in terms of fresh weight (**Figures 1** and **2A–D**) or leaf area (**Figure 2F**). OP alone induced a slight but significant (p < 0.05) increase of the SPAD-index, which has been significantly correlated with chlorophyll concentration, according to absorbance/transmittance measurements (Liu et al., 2017) (**Figure 2E**).

In nitrogen stress conditions (low N), leaf fresh weight, dry weight, and area were decreased by 50%, 47%, and 61%, respectively (**Figures 2A, B, F**) and 31% in roots (**Figures 2A, C**). Plants where visibly chlorotic (**Figure 1**) and SPAD-index values were 81% lower compared to high N controls (p < 0.001) (**Figure 2E**). Also root fresh and dry biomass were similarly affected with a decrease of 31% and 36%, respectively (**Figures 2B, D**). The presence of OP in the growth media had a significant effect in low N conditions by reversing the reduced growth effects of N stress. Growth in terms of fresh weight of shoots and roots was increased by 58% and 71%, respectively in OP treated plants compared to control plants (**Figures 2A, C**). Biomass accumulation was similarly affected with shoot biomass increasing by 61% and root biomass by 68% compared to controls without OP. Leaf area in OP treated plants in low N was 61% larger than control plants (**Figure 2F**). Leaves of OP treated plants in low N were also visibly greener (**Figure 1**) and SPAD-chlorophyll values were fivefold higher than untreated low N plants (<0.001) (**Figure 2E**). Overall, OP treatment did not significantly affect growth in high N conditions, however in low N conditions, it almost completely alleviated the symptoms of N stress induced by 1 mM NO_3_^-^ availability

### Effect of OP on Nitrogen Uptake

In order to determine if OP directly or indirectly affects nitrate uptake, we performed a nitrate uptake assays in increasing concentrations of OP. Our previous findings indicated that high concentrations of OP were inhibitory to growth (Van Oosten et al., 2017). Optimal (1 µM) and higher concentrations of OP were used in a nitrate uptake experiment to determine if high concentrations of OP were affecting nitrate uptake, and therefore, growth. Nitrate uptake of 3-week-old maize roots was assayed in the presence of 1, 10, 50, and 100 µM OP. Roots were incubated with 0, 100, and 500 µM nitrate in the presence and absence of OP. Nitrate uptake was evaluated using a method adapted from Sorgonà et al. (2011) to assess the ability of OP to affect both low-affinity and high-affinity uptake. The inducible low-affinity transport system (LATS) typically functions at concentrations higher than 250 µM NO_3_^-^, whereas the high-affinity transport system (HATS) functions in the range of 10−250 µM NO_3_^-^ (Garnett et al., 2009). Nitrate uptake was affected by OP in a dose dependent manner. At low nitrate concentrations associated with HATS uptake (100 µM NO_3_^-^), OP increased NO_3_^-^ uptake by 30% at 1 µM OP and by 27% at 10 µM OP. Higher OP concentrations had either a reduction effect (50 µM OP) or an inhibitory effect (100 µM OP). OP had a small but significant effect on uptake at higher LATS associated concentrations (500 µM NO_3_^-^), but only at low OP dose (1 µM OP). At concentrations of 10, 50, and 100 µM OP, uptake of 500 µM NO_3_^-^ decreased (**Supplementary Figure 1**).

### ATPase Activity

The ability of OP to act as an inhibitor of membrane bound ATPases was evaluated using 3-week-old root and shoot tissues. The inhibitory effect of OP on ATPases in mammals is well characterized, but the inhibitory effect has not been tested in plants. To evaluate the potential ATPase inhibition in plants by OP, total protein and microsomal membranes were extracted. ATPase activity assays were performed using a dose curve of OP from 0.0001 to 1,000 µM. Sodium-orthovanadate is a strong inhibitor of P-Type ATPases in both plants and animals (Miao and Liu, 2012) and significantly inhibited ATPase activity in all experimental conditions. ATPase in the protein fraction was strongly inhibited by OP at very high concentrations (1 mM) and only in roots (**Supplementary Figure 2**).

### Nitrate and Nitrogen Assimilation in Proteins and Amino Acids

**Figure 3:**
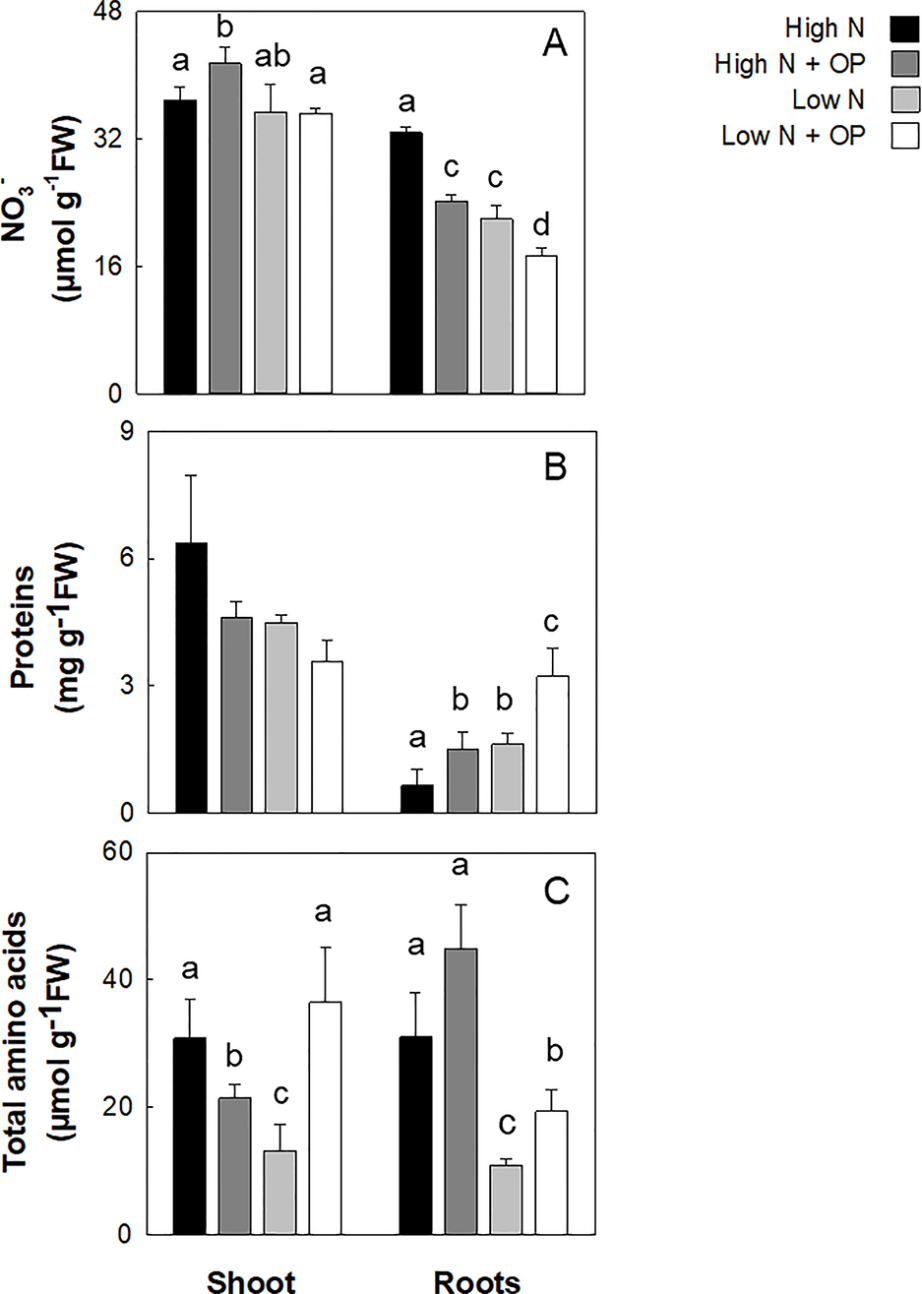
Nitrate **(A)**, protein **(B)**, and total amino acid **(C)** content in maize plants hydroponically grown in control (10 mM NO3-, high N) and stress (1 mM NO3-, low N) nitrogen conditions with and without 1 μM omeprazole (OP). Data are means ± s.d.; n = 3 biological replicates for each treatment. Different letters are significantly different (*p* < 0.05, LSD-test).

Nitrogen assimilation was evaluated through the measurement of NO_3_^-^, total protein content and free amino acids content (**Figure 3**, **Supplemental Table 1**). Nitrate content in untreated high N and low N controls was not statistically significant different in shoots. However, the differences between the two treatments were highly significant in roots (−33%, p < 0.001). In plants treated with OP, NO_3_^-^ content increased in leaves (12%) in high N conditions. Shoots of OP treated plants in low N conditions showed no change in NO_3_^-^ while roots had a significantly lower NO_3_^-^ content (−21%) (**Figure 3**). Protein content was not influenced by N limitation and OP treatment in leaves, while it increased significantly in roots. Low N decreased proteins in control and OP treated leaves of 29% and 44% compared to untreated high N control. On the contrary, low N determined a 1.5- and 3.9-fold increase of protein content in control and OP treated roots, respectively (**Figure 3** and **Table 1**).

**Table 1:** Ions, hydrogen peroxide, malondialdehyde (MDA), carbohydrates, proteins, free amino acids, and polyphenols in shoots and roots of maize plants hydroponically grown in control (high N) and stress (low N) nitrogen conditions with and without 1µM omeprazole (OP). Values are mean ± s.d. (n = 3).

	Shoot																Root															
	High N				High N +OP				Low N				Low N +OP				High N				High N +OP				Low N				Low N +OP			
*Carbohydrates (µmol g* *^-1^* * FW)*																																
Glucose	3.33	±	1.10	^c^	4.22	±	0.53	^c^	5.30	±	0.23	^b^	6.71	±	0.70	^a^	5.05	±	0.37	^bc^	4.83	±	0.22	^c^	6.40	±	1.25	^b^	8.99	±	0.91	^a^
Fructose	2.53	±	0.19	^a^	1.76	±	0.02	^b^	1.93	±	0.63	^ab^	2.76	±	0.94	^a^	2.35	±	0.63	^a^	0.66	±	0.48	^b^	2.20	±	0.66	^ab^	1.35	±	0.25	^b^
Starch	5.57	±	1.52	^c^	11.03	±	2.98	^b^	10.06	±	3.52	^bc^	38.87	±	8.05	^a^	5.67	±	1.13	^c^	10.49	±	3.52	^b^	13.17	±	7.47	^bc^	43.90	±	9.00	^a^
Sucrose	1.12	±	0.10	^c^	0.82	±	0.03	^d^	1.44	±	0.14	^b^	2.29	±	0.23	^a^	1.80	±	0.48	^a^	0.84	±	0.22	^b^	1.17	±	0.13	^b^	2.30	±	0.62	^a^
Soluble proteins (mg g^-1^ FW)																																
Soluble proteins	6.35	±	1.60	^a^	4.61	±	0.37	^a^	4.49	±	0.18	^a^	3.58	±	0.49	^b^	0.65	±	0.38	^c^	1.52	±	0.41	^b^	1.63	±	0.25	^b^	3.22	±	0.67	^a^
*Amino acids (µmol g* *^-1^* * FW)*																																
Total amino acid	30.85	±	5.99	^a^	21.45	±	2.12	^b^	13.20	±	4.10	^c^	36.44	±	8.57	^a^	31.03	±	6.96	^b^	44.97	±	6.76	^a^	10.88	±	0.93	^d^	19.33	±	3.35	^c^
Alanine	1.31	±	0.26	^b^	0.92	±	0.08	^c^	2.07	±	0.70	^b^	7.54	±	1.84	^a^	0.92	±	0.24	^d^	2.94	±	0.42	^b^	1.76	±	0.30	^c^	7.48	±	1.61	^a^
Asparagine	21.88	±	4.35	^a^	14.57	±	1.58	^b^	2.84	±	0.92	^d^	7.97	±	1.77	^c^	17.19	±	4.08	^a^	24.07	±	3.52	^a^	1.29	±	0.17	^b^	1.54	±	0.32	^b^
Aspartate	0.58	±	0.11	^b^	0.43	±	0.03	^c^	0.57	±	0.17	^bc^	1.08	±	0.24	^a^	1.42	±	0.36	^ab^	1.66	±	0.38	^a^	1.00	±	0.10	^b^	1.20	±	0.15	^ab^
BCAAs	0.38	±	0.14	^b^	0.29	±	0.02	^b^	0.34	±	0.05	^b^	0.79	±	0.12	^a^	0.75	±	0.13	^a^	0.87	±	0.09	^a^	0.76	±	0.09	^a^	0.79	±	0.12	^a^
GABA	0.03	±	0.01	^bc^	0.03	±	0.00	^c^	0.04	±	0.01	^b^	0.18	±	0.05	^a^	0.08	±	0.02	^b^	0.12	±	0.03	^ab^	0.04	±	0.01	^c^	0.18	±	0.04	^a^
Glutamate	1.65	±	0.32	^b^	1.12	±	0.07	^c^	1.72	±	0.57	^bc^	3.57	±	0.75	^a^	2.65	±	0.42	^ab^	2.88	±	0.59	^ab^	2.10	±	0.24	^b^	3.22	±	0.41	^a^
Glutamine	0.96	±	0.20	^b^	0.30	±	0.02	^c^	0.69	±	0.23	^b^	2.24	±	0.73	^a^	3.92	±	0.98	^b^	7.81	±	1.08	^a^	1.24	±	0.10	^c^	1.38	±	0.24	^c^
Glycine	1.45	±	0.23	^bc^	1.41	±	0.09	^c^	2.15	±	0.59	^b^	4.26	±	1.03	^a^	0.82	±	0.09	^a^	0.53	±	0.06	^b^	0.23	±	0.03	^c^	0.43	±	0.06	^b^
Minor amino acids	0.80	±	0.22	^b^	0.62	±	0.05	^b^	0.72	±	0.15	^b^	2.35	±	0.41	^a^	1.56	±	0.31	^ab^	2.05	±	0.29	^a^	1.45	±	0.16	^b^	1.48	±	0.25	^b^
Monoethanolamine	0.27	±	0.06	^b^	0.20	±	0.02	^b^	0.24	±	0.09	^b^	0.60	±	0.15	^a^	0.46	±	0.11	^a^	0.43	±	0.10	^a^	0.30	±	0.01	^b^	0.38	±	0.04	^a^
Ornithine	0.24	±	0.05	^a^	0.17	±	0.08	^abc^	0.11	±	0.01	^c^	0.15	±	0.01	^b^	0.90	±	0.15	^a^	0.90	±	0.08	^a^	0.40	±	0.07	^b^	0.32	±	0.03	^b^
Proline	0.30	±	0.06	^ab^	0.34	±	0.03	^ab^	0.30	±	0.01	^b^	0.38	±	0.04	^a^	0.27	±	0.01	^a^	0.33	±	0.06	^a^	0.22	±	0.01	^b^	0.35	±	0.08	^a^
Serine	1.29	±	0.25	^b^	1.27	±	0.11	^b^	1.69	±	0.63	^b^	6.21	±	1.65	^a^	0.71	±	0.17	^b^	1.11	±	0.20	^a^	0.71	±	0.06	^b^	1.19	±	0.22	^a^
Threonine	0.09	±	0.02	^b^	0.07	±	0.02	^bc^	0.05	±	0.02	^c^	0.49	±	0.15	^a^	0.15	±	0.04	^ab^	0.22	±	0.04	^a^	0.16	±	0.02	^b^	0.18	±	0.04	^ab^
*Ions and hydrogen peroxide (µmol g* *^-1^* * FW)*																																
Acetate	3.48	±	0.30	^a^	3.10	±	0.37	^a^	3.27	±	0.72	^a^	1.95	±	0.18	^b^	6.05	±	0.33	^a^	5.76	±	0.68	^a^	4.41	±	0.62	^b^	4.32	±	0.20	^b^
Chloride	62.51	±	2.95	^a^	52.53	±	2.11	^b^	72.69	±	16.06	^a^	48.63	±	1.59	^c^	32.08	±	3.54	^b^	29.27	±	1.62	^b^	41.95	±	4.41	^a^	42.83	±	6.09	^a^
Malate	2.32	±	0.12	^c^	1.64	±	0.07	^d^	3.85	±	0.93	^a^	2.73	±	0.05	^b^	1.05	±	0.10	^c^	0.64	±	0.11	^d^	2.09	±	0.25	^b^	2.74	±	0.18	^a^
Nitrate	36.92	±	1.56	^b^	41.51	±	1.94	^a^	35.34	±	3.48	^b^	35.19	±	0.74	^b^	32.76	±	0.72	^a^	24.18	±	0.78	^b^	21.93	±	1.70	^b^	17.38	±	1.00	^c^
Oxalacetate	2.16	±	0.10	^a^	1.97	±	0.25	^a^	0.93	±	0.14	^c^	1.21	±	0.12	^b^	4.65	±	0.42	^a^	3.78	±	0.23	^b^	3.96	±	0.64	^ab^	3.57	±	0.13	^b^
Sulphate	1.82	±	0.08	^b^	2.02	±	0.03	^a^	2.07	±	0.32	^ab^	1.28	±	0.03	^c^	7.84	±	0.60	^c^	7.08	±	0.45	^c^	12.26	±	1.07	^a^	10.57	±	0.16	^b^
Hydrogen peroxide	3.10	±	0.66	^c^	3.23	±	0.31	^c^	7.38	±	0.64	^a^	4.70	±	0.34	^b^	2.74	±	0.34	^a^	2.70	±	0.57	^a^	2.63	±	0.23	^a^	1.81	±	0.23	^b^
Other compounds																																
MDA (nmol g^-1^ FW)	28.70	±	0.51	^b^	34.32	±	4.93	^a^	24.12	±	3.50	^c^	28.45	±	0.87	^bc^	81.00	±	10.67	^a^	83.91	±	8.57	^a^	51.23	±	5.75	^b^	48.78	±	2.32	^b^
Polyphenols (mg_GAE_ g^-1^ FW)	2.34	±	0.19	^a^	1.99	±	0.08	^b^	2.35	±	0.23	^a^	1.74	±	0.13	^c^	1.15	±	0.13	^a^	1.35	±	0.18	^a^	1.34	±	0.21	^a^	1.32	±	0.06	^a^

Means in the same rows with different letters are significantly different (p < 0.05, Student's t-test).

The total amino acid concentration of shoots and roots of high N plants was differently affected by OP. While in leaves OP decreased significantly (p < 0.05) its content by 30% compared to the untreated shoots, in roots OP increased the amino acids content by 45% (p < 0.05). Total amino acids were strongly influenced by N limitation and OP treatment with significant low N × OP interaction. Low N decreased free amino acids of 61%, on average, in both organs (p < 0.01); low N + OP determined the opposite effect, with a significant increase (p < 0.01) of 176% and 78% in shoots and roots, respectively, compared to the low N without OP.

Asparagine was quantitatively the major amino acid representing about 71% and 55% of total free amino acids in shoots and roots of high N plants, respectively (**Table 1**). Low nitrate strongly affected this amino acid content, decreasing it to 13% and 7% of the high N values in shoots and roots, respectively. High N + OP had the same effects on shoots and roots total amino acids (probably because of the high asparagine contribution to the total amino acids content); while at low N, OP strongly increased asparagine content only in leaves (+2.8-fold, p < 0.01). This same effect induced by low N × OP treatment in shoots was also observed for aspartate (+ 90%), glutamine, glutamate (+ 110%), monoethanolamine (MEA) (+ 150%), glycine (+ 100%), serine (+ 270%), threonine (+ 860%), minor amino acids (+ 230%), and, specifically, branched chain amino acids (BCAAs) which belong to minor amino acids (+ 130%) (**Table 1**). Alanine and GABA underwent a very strong increase in their content under low N + OP, both in shoots and roots compared to low N controls (**Table 1**). Alanine increased by 3.6- and 4.3-fold in shoots and roots, respectively; while GABA showed a 4.3- and 5.2-fold increase in shoots and roots, respectively.

### OP Effects on NR Activity and Activation State

**Figure 4:**
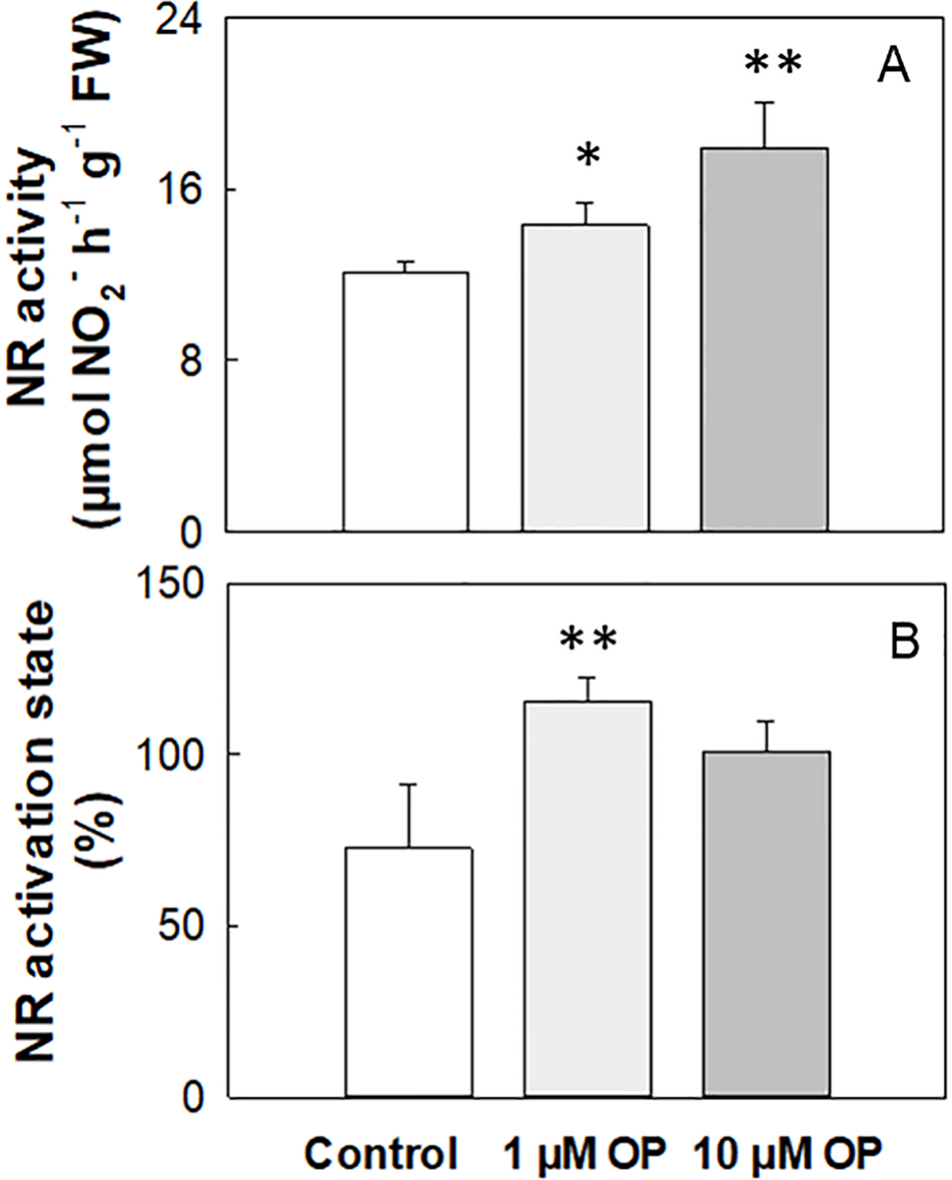
Nitrate reductase activity **(A)** and activation state **(B)** of shoots from maize (*Zea mays* L.) plants hydroponically grown in control (10 mM NO_3_^-^, high N) and stress (1 mM NO_3_^-^, low N) nitrogen conditions with (black bars) and without 1 µM omeprazole (OP) (white bars). Data are means ± SD; n = 3 biological replicates for each treatment. Asterisks denote significant differences (*, P < 0.05; **, P < 0.01) between different OP treated plants.

Total NR activity in maize shoots and roots of shock frozen and stored at −80°C plant material was 4.02 and 4.22 µmol NO_2_^-^ h^-1^ g^-1^ FW, respectively (**Table 2**). The activation state strongly increased under OP treatment independently of N nutrition and organ, even if not significantly in shoots (**Table 2**). On the contrary, in fresh harvested plant shoots under high N the NR activity was on average 12.14 µmol NO_2_^-^ h^-1^ g^-1^ FW, and it significantly increased (p < 0.01) of 18% and 48% under 1 and 10 µM OP, respectively (**Figure 4A**). The activation state of fresh harvested control shoots was 72.8%, and the OP treatment increased this value of 60% and 39% under 1 and 10 µM OP, respectively (**Figure 4B**).

**Table 2:** Carbon and nitrogen metabolism enzymes and antioxidant enzymes activities in shoots and roots of maize plants hydroponically grown in control (high N) and stress (low N) nitrogen conditions with and without 1 µM omeprazole (OP). Values are mean ± s.d. (n = 3).

	Shoot																Root															
	High N				High N +OP				High N				Low N +OP				High N				High N +OP				Low N				Low N +OP			
*Carbon and nitrogen metabolism enzymes (µmol h* *^-1^* * g* *^-1^* * FW)*																																
AGPase	39.82	±	6.49	^a^	40.17	±	3.24	^a^	11.53	±	3.09	^c^	26.33	±	3.33	^b^																
C-Citrate synthase	27.71	±	5.97	^a^	26.01	±	4.97	^a^	20.29	±	5.81	^ab^	15.92	±	4.80	^b^	45.20	±	6.51	^a^	46.00	±	5.04	^a^	51.09	±	4.86	^a^	41.72	±	10.70	^a^
M-Citrate synthase	19.01	±	3.05	^a^	15.88	±	4.74	^ab^	11.54	±	1.76	^b^	14.12	±	3.47	^ab^	33.00	±	5.27	^a^	33.83	±	3.37	^a^	35.43	±	3.12	^a^	31.14	±	3.99	^a^
G6P DH	15.76	±	2.01	^a^	14.94	±	4.85	^ab^	12.71	±	3.91	^ab^	10.28	±	3.18	^b^	24.33	±	3.91	^a^	23.55	±	2.76	^a^	24.96	±	4.48	^a^	21.36	±	2.06	^a^
Glucokinase	4.84	±	1.26	^a^	3.79	±	0.23	^a^	5.29	±	1.28	^a^	4.95	±	1.08	^a^	8.47	±	0.46	^a^	9.10	±	0.42	^a^	8.21	±	1.60	^a^	10.61	±	1.95	^a^
Glutamate DH (NAD)	11.44	±	2.60	^ab^	11.02	±	0.55	^a^	5.29	±	1.55	^b^	7.07	±	2.11	^b^	45.59	±	7.31	^a^	29.69	±	2.88	^b^	25.69	±	7.90	^b^	17.78	±	3.43	^c^
Isocitrate DH (NAD)	5.85	±	0.96	^a^	4.64	±	1.13	^a^	5.33	±	1.17	^a^	6.27	±	4.21	^a^	14.54	±	3.40	^a^	18.93	±	3.19	^a^	24.36	±	7.26	^a^	18.35	±	4.37	^a^
Isocitrate DH (NADP)	9.77	±	1.43	^a^	9.17	±	1.21	^a^	7.77	±	2.16	^a^	7.57	±	0.81	^a^	67.00	±	6.48	^a^	63.12	±	5.41	^a^	58.00	±	8.47	^a^	64.35	±	13.35	^a^
NR activity	4.37	±	0.87	^a^	3.38	±	0.48	^a^	4.61	±	1.85	^a^	3.86	±	0.92	^a^	3.87	±	1.59	^a^	4.20	±	1.42	^a^	4.65	±	1.88	^a^	4.19	±	0.64	^a^
NR Activation state (%)	65.1	±	24.5	^a^	114.4	±	34.9	^a^	68.6	±	24.8	^a^	118.2	±	44.4	^a^	79.2	±	22.8	^ab^	116	±	22	^a^	77.1	±	9.9	^b^	113	±	27	^ab^
PEP Carboxylase	145	±	29	^ab^	187	±	51	^a^	104	±	32	^ab^	63.4	±	16.7	^b^	25.8	±	4.3	^a^	29.5	±	6.3	^a^	29.8	±	7.1	^a^	32.8	±	8.4	^a^
Pyruvate kinase	171	±	15	^a^	166	±	5	^a^	118	±	13	^b^	142	±	29	^ab^	49.2	±	9.8	^a^	53.1	±	11.0	^a^	59.5	±	14.8	^a^	50.8	±	9.7	^a^
*Antioxidant enzymes (mmol h* *^-1^* * g* *^-1^* * FW)*																																
APX	1.08	±	0.19	^b^	1.67	±	0.16	^a^	1.87	±	0.26	^a^	1.57	±	0.70	^ab^	1.12	±	0.30	^b^	1.93	±	0.34	^a^	1.22	±	0.35	^b^	0.92	±	0.03	^b^
CAT	1.59	±	0.29	^a^	1.93	±	0.12	^a^	1.89	±	0.03	^a^	1.67	±	0.23	^a^	1.49	±	0.41	^b^	1.93	±	0.40	^ab^	1.74	±	0.19	^b^	2.03	±	0.10	^a^
GR	1.52	±	0.18	^ab^	1.58	±	0.03	^a^	1.37	±	0.10	^b^	1.51	±	0.08	^ab^	1.61	±	0.13	^b^	1.67	±	0.19	^ab^	1.67	±	0.21	^ab^	1.80	±	0.07	^a^
SOD	4.49	±	1.65	^a^	4.01	±	1.77	^a^	4.86	±	0.52	^a^	2.99	±	2.22	^a^	4.21	±	1.51	^a^	4.41	±	0.65	^a^	3.84	±	1.64	^a^	3.20	±	0.64	^a^

**Figure 5:**
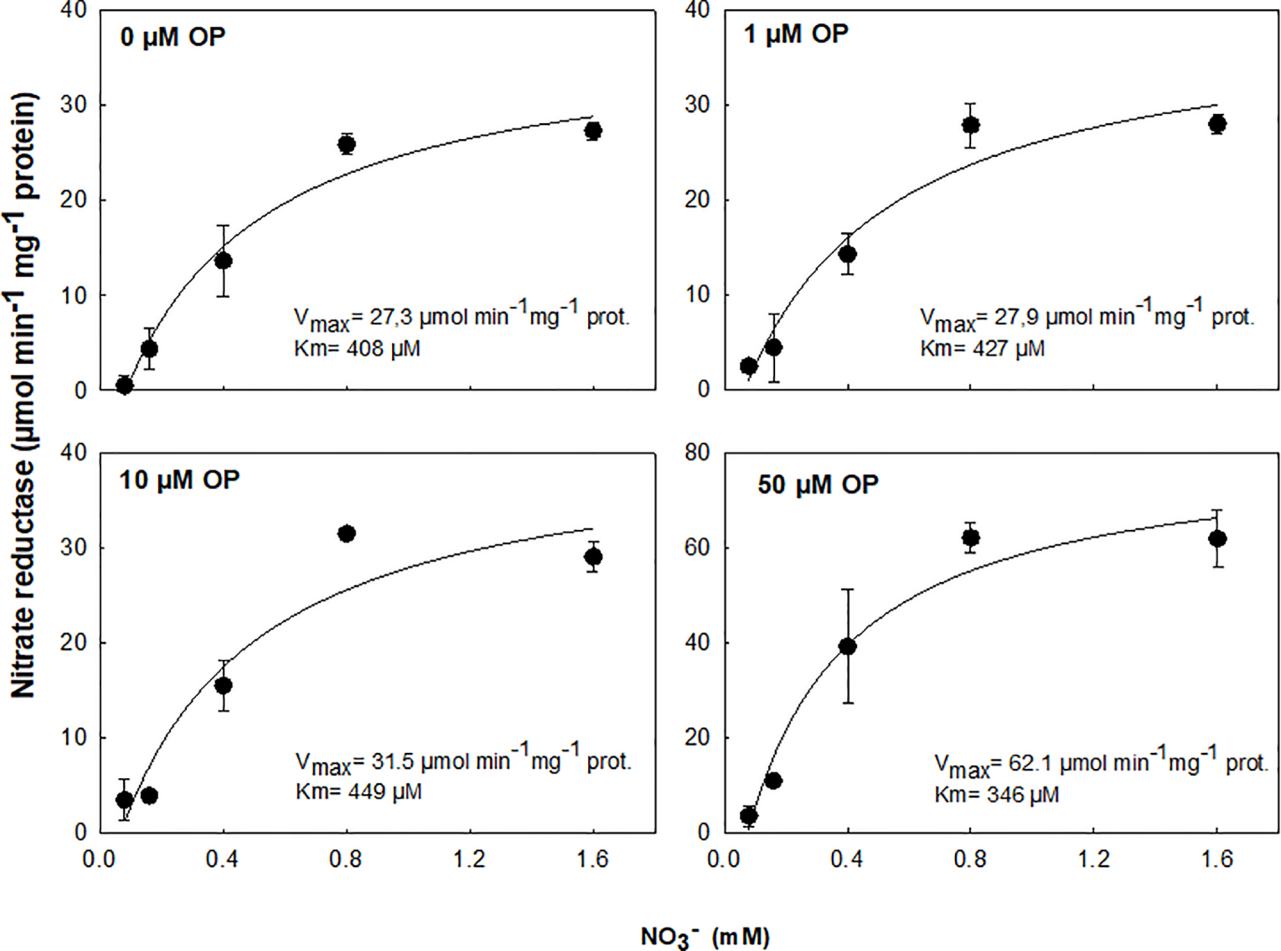
*In vitro* activity assay of purified NADH-Nitrate Reductase (NR) from *Arabidopsis thaliana* in cell-free extracts with 0.08, 0.16, 0.4, 0.8, and 1.6 mM NO_3_^-^ and 0, 1, 10, or 50 µM omeprazole (OP). Data are means ± s.d. (shown by vertical bars when larger than the symbol); n = 3 from independent experiments. Lines were fitted by linear regression.

The effect of OP treatment was tested on purified protein from Arabidopsis. OP 50 µM was able to increase the enzyme catalytic efficiency and the specificity for NO_3_^-^ (as substrate) resulting in an increased V_max_ and decreased K_m_ (**Figure 5**). This suggests that OP helps in maintaining adequate affinity of enzyme toward its substrate as well as its catalytic rate though a possible physical interaction with NR.

### Carbohydrates, Starch, and Citric Acid Cycle Content

Starch content was equivalent in shoots and roots under high N treatment (5.6 µmol G_eq_ g^-1^ FW on average) and low N (11.6 µmol G_eq_ g^-1^ FW on average). OP treatment resulted in an increase of 2.0- and 1.8-fold of starch content in high N shoots and roots, respectively; while a much stronger effect was exerted at low N with 3.9- and 3.3-fold increases in the two organs, respectively (**Table 1**). Sucrose levels were decreased in OP treated high N shoots (−30%, p < 0.01) and roots (−50%, p < 0.05). A strong increase was observed for sucrose and hexoses under OP treated low N conditions (p < 0.05). OP treatment caused a shift in primary metabolism that shunted sugars into starch storage in both low and high N conditions. In shoots under low N and OP conditions, this was a consequence of a 2.8-fold increase of ADP-glucose pyrophosphorylase (AGPase) activity (**Table 2**).

OP treatment also perturbed the citric acid cycle. Levels of oxaloacetate and malate were evaluated (**Table 1**). Oxaloacetate was 2.1 and 4.2 µmol g^-1^ FW in shots and roots of high N plants, respectively, independently of OP. OP significantly decreased (−40%, p < 0.01) acetate content only in low N shoots. Malate was 2.3 and 3.9 µmol g^-1^ FW in shots of control high and low N plants, respectively; while it was 45% and 54% lower in control high and low N roots compared to relative shoots. OP reduced malate content in shoots both high N and low N (−29%); but it had an opposite effect on roots, decreasing its content at high N (−39%) while increasing it at low N (+31%) (**Table 1**). Acetate, even not belonging to the citric acid cycle, had a similar trend to that of oxaloacetate, with the difference that low N did affect control roots (−27%, p < 0.05) but not shoots (**Table 1**).

### OP Effects on Hydrogen Peroxide, MDA, Polyphenols, and Antioxidant Enzymes

Formation of hydrogen peroxide (H_2_O_2_) under nitrogen deficiency is indicative of reduced efficiency of electron transport systems and can act as a redox signal (Kandlbinder et al., 2004; Tewari et al., 2007). Nitrogen stress induced formation of H_2_O_2_. Its concentrations in low N controls were 7.38 µmol g^-1^ FW in shoots and 2.63 µmol g^-1^ FW in roots. The H_2_O_2_ content in low N shoots was 2.38-fold higher than the high N controls. OP reduced H_2_O_2_ content by 34% in roots and shoots (**Table 1**). Malondialdehyde (MDA) content was 69% higher in shoots and 185% higher in roots under low N conditions. However, OP treatment had no effect on the MDA concentration (**Table 1**).

Polyphenols play a key role in antioxidant activity and membrane protection in plants (Woodrow et al., 2017). Their content was, on average, 2.34 and 1.24 mg GAE g^-1^ FW in shoots ad roots, respectively. OP had no effect on roots, while it significantly decreased the content of these metabolites both in high N shoots (−15%, p < 0.05) and low N shoots (−26%, p < 0.01) (**Table 1**).

Catalase (CAT), glutathione reductase (GR), and superoxide dismutase (SOD) activities were fairly constant independently of N nutrition, OP treatment and organ, being, on average 1.8, 1.6, and 4.0 U g^-1^ FW, respectively (**Table 1**). On the contrary, ascorbate peroxidase (APX) varied in dependence on N nutrition and OP treatment. In high N control shots and roots, it was 1.1 U g^-1^ FW. Low N increased APX activity in shoots (+73%) but not in roots. OP significantly affected this enzyme activity only in plants grown under high N, increasing it of 54 (p < 0.01) and 72% (p < 0.05) in shoots and roots, respectively, compared to controls (**Table 1**).

### Gene Expression

**Figure 6:**
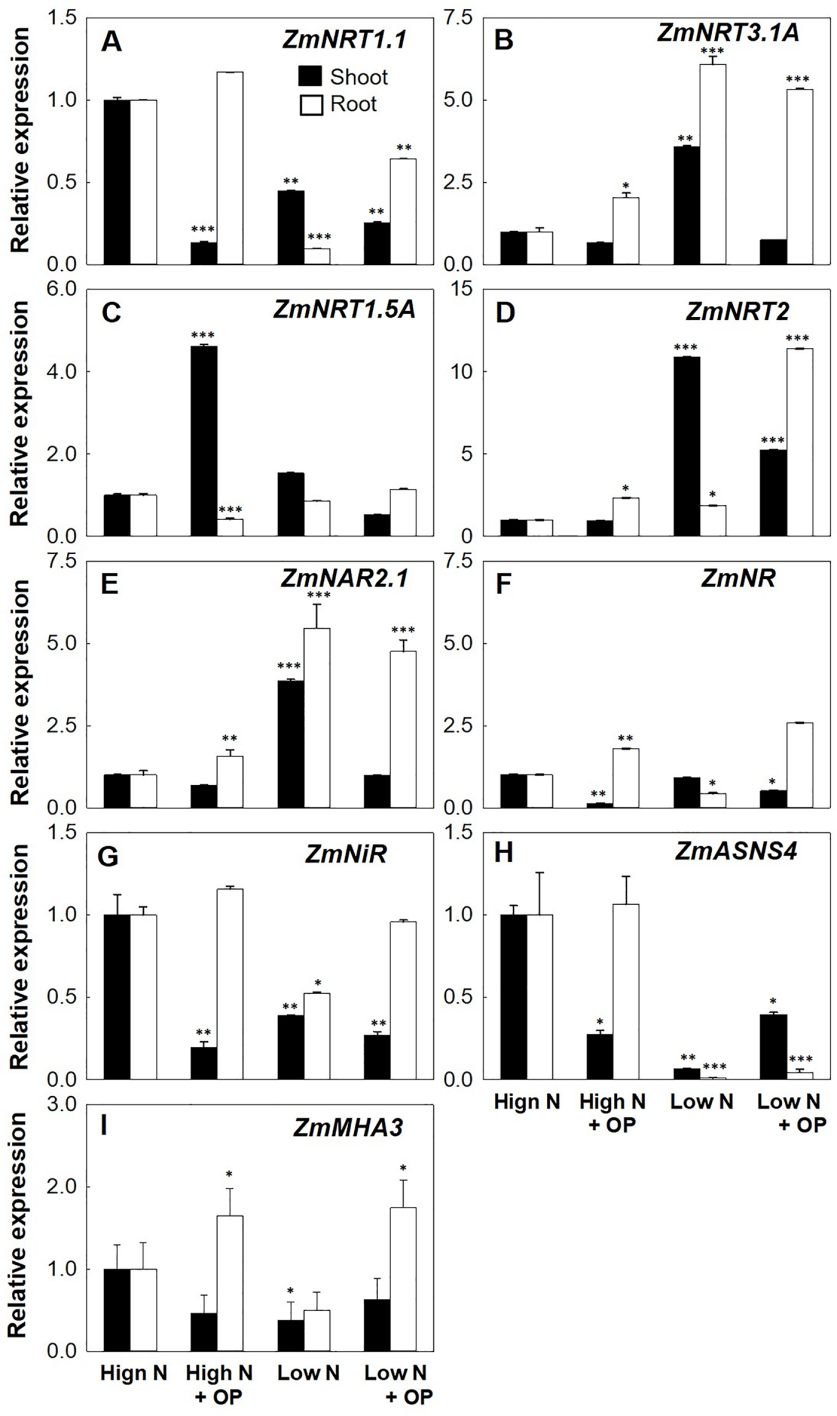
Nitrogen uptake and assimilation genes expression of maize plants hydroponically grown in in control (10 mM NO_3_^-^, high N) and stress (1 mM NO_3_^-^, low N) nitrogen conditions with and without 1 µM omeprazole (OP) in shoot (black bars) and root (white bars). Relative gene expression (in terms of fold change) of **(A)** nitrate transporter 1.1 *ZmNRT1.1*, **(B)** nitrate transporter 1.3A *ZmNRT1.3A*, **(C)** nitrate transporter 1.5A *ZmNRT1.5A*, **(D)** nitrate transporters 2.1 and 2.2 *ZmNRT2*, **(E)** NAR2-like partner protein *ZmNAR2*.1, **(F)** nitrate reductase *ZmNR*, **(G)** nitrite reductase *ZmNiR*, **(H)** asparagine synthetase *ZmASN4*, and **(I)** the plasma membrane MHA3 ATPase *ZmMHA3*. Data are means of relative expression measurements based on the ∆∆Ct Method ± s.d.; n = 3 biological replicates for each treatment. Asterisks denote significant differences (*, P < 0.05; **, P < 0.01; ***, P < 0.001) between different each treatment and controls under high N according to Student’s T-Test.

*NRT2.2* and *NRT2.1* are the main genes controlling the high-affinity nitrate uptake in maize (Garnett et al., 2013). OP treatment increased the root expression of *ZmNRT2.1* of threefold in high N and almost fourfold in low N conditions. Shoot expression of *ZmNRT2.1* was not significantly influenced by OP in high N conditions while it was half of untreated controls in low N conditions (**Figure 6A**). The *ZmNAR2.1* and *NAR2*-like gene *ZmNRT3.1A* coding for accessory protein complexes with NRT2.1, implicated in NRT2.1 localization or stabilization at the PM as well as involved in iHATS signaling (Okamoto et al., 2006; Yong et al., 2010; Chen et al., 2017), were highly induced by OP. Low N conditions induced the expression of these two genes in both roots and shoots, with OP inducing a twofold increase in high N conditions. Similar to *ZmNRT2.1* expression in low N with OP, *ZmNAR2.1* and *ZmNRT3.1A* were decreased in shoots under low N and OP. *ZmNPF6.3/NRT1.1,* which is expressed in root tissues as well as in young shoot tissues and is involved in nitrate uptake from soil and its translocation to shoot (Noguero and Lacombe, 2016) and that can functions also as nitrate sensor promoting and controlling root system architecture (Medici and Krouk, 2014), was highly increased by OP in particular in root both under high N and low N. Also *ZmNPF7.3/NRT1.5*, coding for a low-affinity bidirectional nitrate transporter involved in xylem loading and root-to-shoot translocation of nitrate (Zheng et al., 2016), was highly expressed in leaves of high N plants under OP treatment, too.

Cytosolic NRs reduce nitrate translocated from the roots into nitrite and are controlled transcriptionally and posttranslationally (Krapp, 2015). Low N conditions resulted in a down regulation of *ZmNR* in roots (**Figure 6H**). OP treatment reduced the expression of NR in shoots for both low and high N conditions. In roots, OP treatment significantly increased NR expression twofold in high N conditions and 2.5-fold in low N conditions. Nitrite reductase gene (*ZmNiR*) demonstrated a similar pattern of expression under OP treatment with reduced expression in shoots and increased expression in roots (**Figure 6I**). The asparagine synthetase ZmASN4 is one of four isoforms in maize responsible for N assimilation. Asparagine is the amide having the higher N/C ratio (2N:4C) and as the major form of transport for N in plants is involved in assimilation, distribution, and remobilization (Krapp, 2015). In low N condition *ZmASN4* was severely down regulated in roots and shoots. *ASNS4* is ubiquitously expressed in all tissues and is transcriptionally downregulated in maize under nitrogen starvation (Todd et al., 2008; Zanin et al., 2015). In low N plants treated with OP, shoot expression was higher. Interestingly, under high N and OP treatment, shoot expression of *ZmASN4* was significantly downregulated, likely indicating a negative feedback regulation.

### PCA, Heat Map, and Correlation Analyses

**Figure 7:**
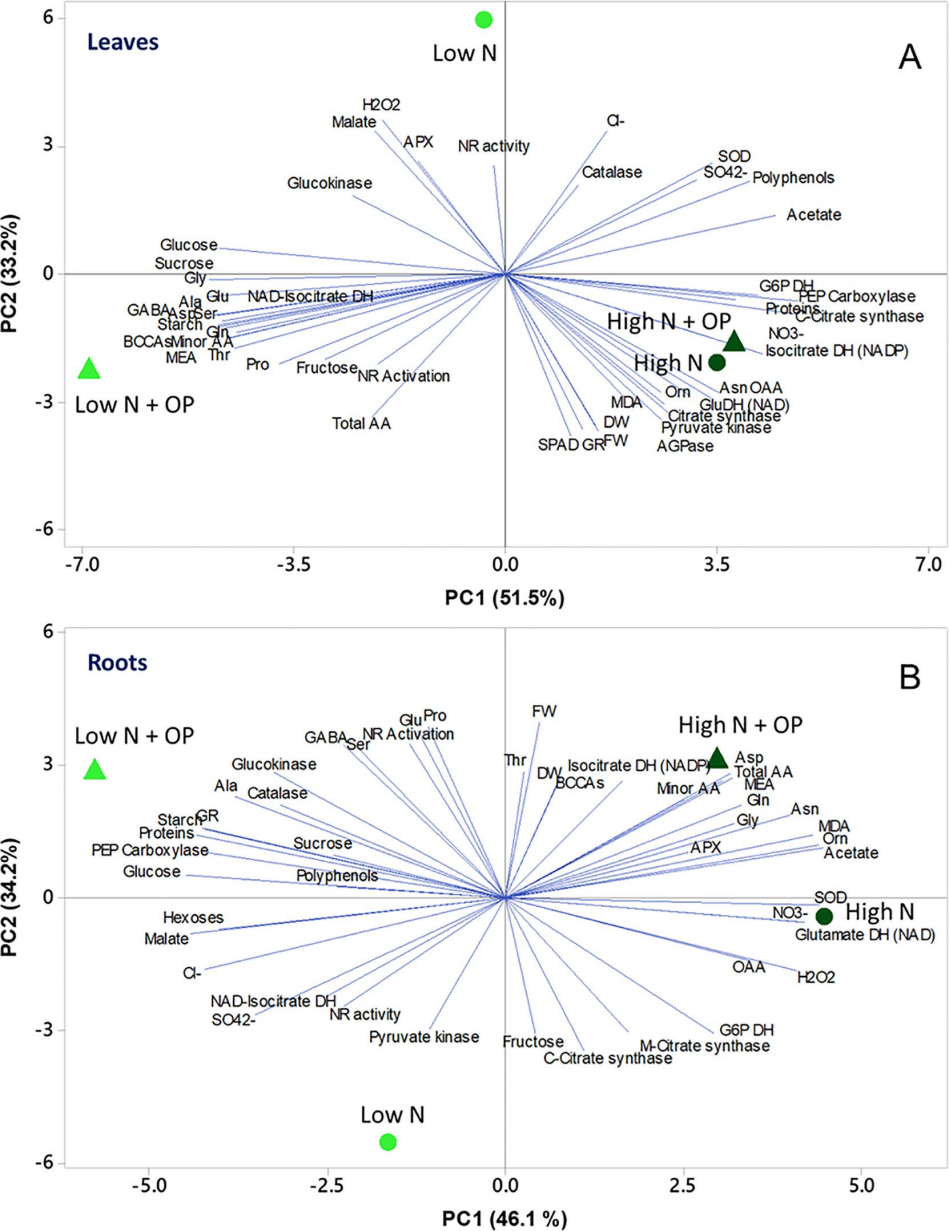
Principal component analysis (PCA) scatter plots based on the first two principal components (PC1 and PC2) generated from the analyzed physiological parameters in maize plants for shoot **(A)** and root **(B)** under control (10 mM NO_3_^-^, high N) and stress (1 mM NO_3_^-^, low N) nitrogen conditions with and without 1 µM omeprazole (OP).

To obtain an overview of the effects of OP × nitrogen nutrition on growth and biochemical parameters of maize seedlings, a PC analysis (PCA) was carried out. In shoots, the first two PCs were related with Eigen values > 1 and explained 84.6% of the total variance with PC1 and PC2 accounting for 51.5% and 33.2%, respectively (**Figure 7A**). PC1 was positively correlated to nitrate, sulphate, asparagine, oxaloacetate and acetate, proteins, fresh and dry weight, enzymatic activities, and polyphenols. PC1 was also negatively correlated to amino acids, in particular alanine, serine, minor amino acids, GABA, glutamate and glutamine, MEA, starch, and soluble sugars. Moreover, PC2 was positively correlated to hydrogen peroxide, malate, APX, catalase, chloride, and NR activity. Furthermore, the PCA scatter-plot split the samples into three main groups, with high N and high N + OP clustered together clustered in the fourth quadrant in the positive side of PC1, completely separated from the other two treatments. They showed the highest nitrate and chlorophyll content (SPAD), but also the highest MDA concentration and GR and other carbon enzymes activities. Low N was clustered in the second quadrant, in the negative side of PC2, showing the lowest fresh weight, dry weight, chlorophyll content but also the lowest MDA content (**Figure 7A**). Low N + OP cluster in the third quadrant, in the negative side of PC1, had the highest total and minor amino acid, included glutamate, glutamine, aspartate, GABA, and MEA, in addition to starch and soluble sugars content.

In roots the first two PCs were related with Eigen values > 1 and explained 80.3% of the total variance. Nitrogen nutrition contributed to the clear separation on PC1, which described 46.1% of the variability, while the treatment with OP contributed to separation on PC2, which described 34.2% of the variability (**Figure 7B**). PC1 was positively correlated to total amino acids, in particular amides and ornithine, but also MDA, SOD, and GDH activities; while it was negatively correlated to glucose, malate, soluble proteins, starch, sulphate and chloride, GR, and PEPC activities. Moreover, PC2 was positively correlated to fresh weight and dry weight, proline, glutamate, NR activation, GABA, BCAAs, serine, and threonine; while it was negatively correlated to fructose, mitochondrial and cytosolic citrate synthase activities, pyruvate kinase, and NR activities. The score plot of the PCA divided the four treatments in different quadrants with high N and high N + OP, in the fourth and first quadrant, respectively, but without a sharp division between the two treatments. High N showed the highest nitrate and OAA, but also the highest hydrogen peroxide and SOD activities; while high N + OP showed the major total amino acid content, in particular glutamine, aspartate, and minor amino acids. Low N + OP was clustered in the second quadrant, in the negative side of PC1, and showed the major carbohydrate and protein content, glutamate, proline and GABA content, and the highest PEPC, catalase, and GR activities. Low N was clustered in the third quadrant, in the negative side of PC2, and showed the highest sulphate content and NR activity, but also the lowest amino acid content, dry and fresh weight (**Figure 7B**).

**Figure 8:**
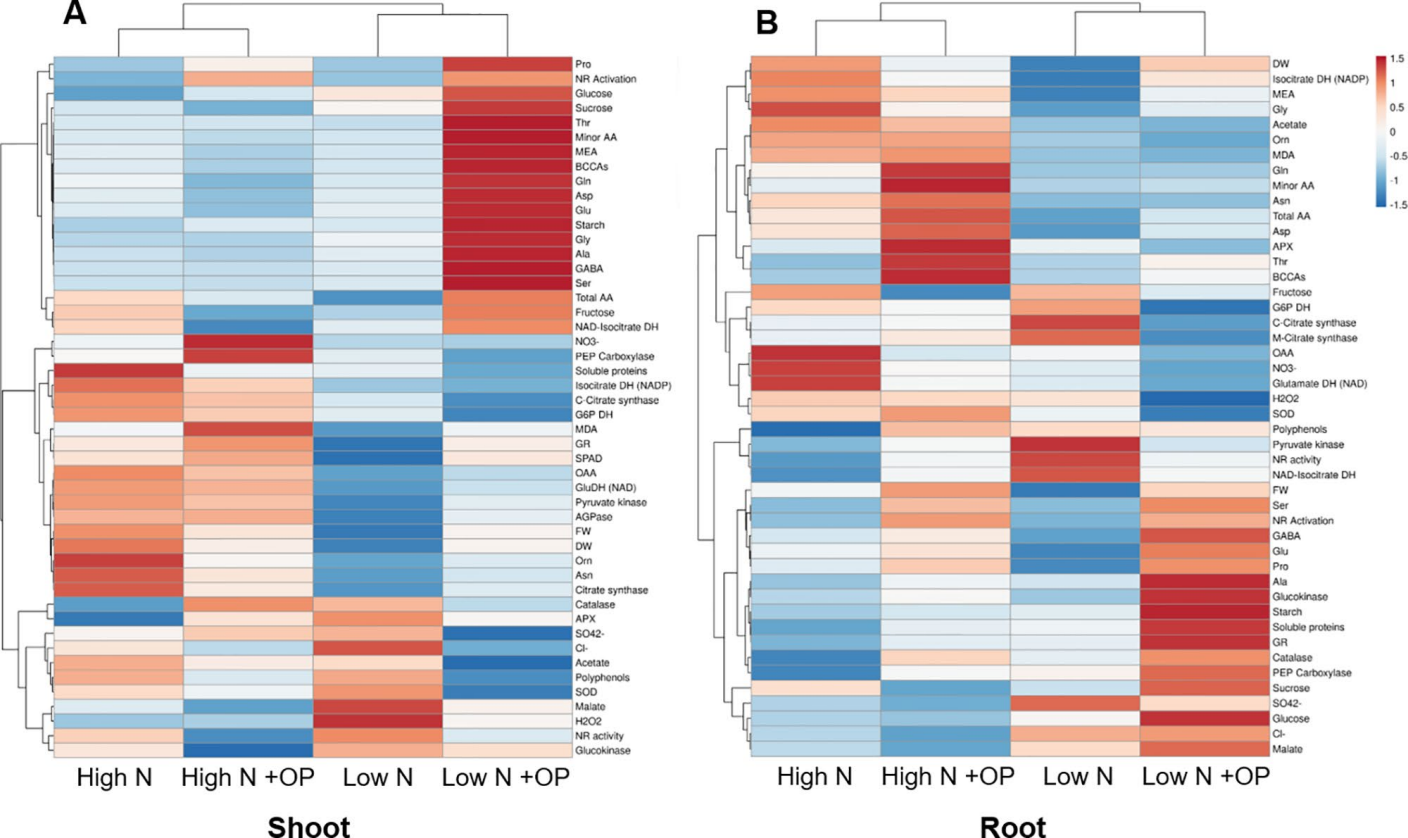
Cluster heat map analysis summarizing the maize shoot **(A)** and root **(B)** responses to high and low nitrogen concentration in the nutrient solution and omeprazole (OP) treatment. Plants were hydroponically grown in control (10 mM NO_3_^-^, high N) and stress (1 mM NO_3_^-^, low N) nitrogen conditions with and without 1 µM OP. The heat map was generated using the https://biit.cs.ut.ee/clustvis/online program package with Euclidean distance as the similarity measure and hierarchical clustering with complete linkage. Data presented are means of biological replicates; biometric measurements (FW of shoots, DW of shoots, FW of roots, DW of roots, leaf area) (n = 6), SPAD index (n = 20), and other biochemical measurements (n = 3).

A heat map providing the biochemical and physiological changes of maize plants in response to nitrogen × OP was displayed in **Figure 8**. The heat-map identified two main clusters in both shoot and root, with nitrogen being the main clustering factor in both shoots and roots, followed by OP. In particular, high N treatments clustered separated from the other two low N treatments in shoots because of their higher fresh and dry weight, SPAD index, asparagine, oxaloacetate, carbon enzymes activity and nitrate content (**Figure 8A**), and in roots because of their higher MEA, glycine, acetate, ornithine, MDA, total amino acid content, in particular asparagine and aspartate (**Figure 8B**). Indeed, two separated subclusters could be defined under both the first and the second clusters which illustrated the nitrogen × OP interaction. In particular, the OP application at high N in the shoots separated from that with high N without OP because of the higher nitrate, SPAD index, PEPC, but also the highest MDA, catalase and GR activity, and lowest sugar content and NR activity (**Figure 8A**). While low N + OP subclustered separately from low N without OP in the shoots because of the highest total and minor amino acid content, with the exception of asparagine, highest carbohydrate content, and lowest protein and carbon metabolism enzymes activity (**Figure 8A**). In the roots, low N + OP clustered separately from low N without OP because of the highest carbohydrate and protein content, PEPC, glucokinase and GR activities, GABA, alanine, glutamate, and proline content (**Figure 8B**).

The correlation analysis showed in roots a negative correlation between the level of nitrate and soluble proteins (r = 0.86; p < 0.001), but a positive correlation between fresh weight and free amino acids (r = 0.73; p < 0.001). Particularly in roots under low N, there was a negative correlation between the level of asparagine and soluble proteins (r = 0.75; p < 0.05), but a positive one between malate and free amino acids (r = 0.85; p < 0.05) and glutamate and soluble proteins (r = 0.91; p < 0.05).

## Discussion

The targets and effects of OP in mammals have been well characterized for many decades (Clissold and Campoli-Richards, 1986; Massoomi et al., 1993). Recent reports have established that OP applications in plants elicit two major phenotypes: growth promotion and salt tolerance (Van Oosten et al., 2017a; Rouphael et al., 2018; Carillo et al., 2019d; Cirillo et al., 2019). Recently it has been proved that also OP can enhance mechanical stress tolerance (Okamoto et al., 2018). In this study, we further investigated if mechanisms directly involved with nitrate uptake and assimilation could be perturbed or activated by OP treatment contributing to the growth promotion phenotype. Previous experiments indicated that OP treatment increased nitrate content in tomato (Van Oosten et al., 2017a; Rouphael et al., 2018), basil (Carillo et al., 2019a; Carillo et al., 2019d), and in *Arabidopsis thaliana* (unpublished results). This led to the hypothesis that OP may affect the uptake and assimilation of nitrogen thus enhancing growth. Our results indicate that OP does indeed enhance NUE through nitrogen assimilation and this small molecule also perturbs several primary and secondary metabolic pathways. Here, we have characterized the primary phenotype under N stress with OP and many of the metabolites that are altered upon treatment. Furthermore, we have evidence that OP directly interacts with NR, enhancing assimilation through an increased affinity for the substrate and constitutive activation of the enzyme.

### Nitrogen Stress and Omeprazole Treatment

While we did not observe a significant growth enhancement in maize by OP at 1 µM, we did observe that it alleviates much of the growth penalty imposed by severely limiting available nitrogen in the nutrient solution and is reflected in the fresh and dry weights of maize leaves and roots (**Figure 1**). Generally, OP treatment almost completely restored growth in N-limiting conditions (**Figures 1A–D**).

In plants grown under low N, the free amino acids decreased both in shoots and roots, while proteins decreased in shoots and increased in roots compared to controls. OP appeared to further decrease the content of proteins in the leaves while stimulating the synthesis of free amino acids, in particular glutamine and asparagine and their transport to the roots. The two amides are used by the plant as a long-distance nitrogen transport molecule, given their high N/C ratio. When formed, they can be loaded into the phloem and sent to the roots to be converted to glutamate and then used as amine donor for the synthesis of all other protein amino acids.

In addition, both in shoots and roots alanine and GABA strongly increased under low N × OP treatment. This could be an important mechanism of biochemical pH-stat for the viability of maize plant under N limitation. In fact, the synthesis of alanine trough the decarboxylation of malate to pyruvate operated by malic enzyme and the decarboxylation of glutamate to GABA by mean of glutamate decarboxylase are proton-consuming reactions buffering cytosolic acidosis (Limami et al., 2008; Carillo, 2018). Moreover, alanine transaminated to pyruvate can be converted to acetyl-Coenzyme A in the mitochondrial matrix, while GABA shunt can provide NADH and/or succinate to tricarboxylic acid (TCA) cycle exerting an anaplerotic function (Bouché and Fromm, 2004) that sustains plants under N stress (Hare and Cress, 1997; Carillo, 2018). Therefore, OP induced GABA and alanine synthesis under low N could be crucial to ensure plant viability under this abiotic stress, supplying carbon skeletons and ATP for enhancing the synthesis of amino acids and proteins especially in the roots (Carillo et al., 2019b). This may increase root growth and improve the absorption and assimilation of nutrients from the soil. Accordingly, OP treatment significantly decreased nitrate content in the roots compared to the controls. This result is consistent with the reported role of GABA and alanine in stress mitigation against abiotic stresses (Michaeli and Fromm, 2015; Diab and Limami, 2016; Carillo, 2018). However, it is not excluded that OP influence on GABA and alanine concentrations may just be a downstream consequence of the increased N uptake or mobilization. In fact, as previously seen for amides, it seems that OP favors mechanisms of mobilization of the N resources already present in the shoot tissues to synthesize new amino acids to transport to root. This is probably obtained by diverting the available energy from protein or polyphenols synthesis to the synthesis of new amino acids in shoots. Moreover, it is likely that exists a trade-off between polyphenols and ROS, which represent energy, and carbon plus nitrogen metabolism as indicated by PCA. OP seems to favor carbon and nitrogen metabolism. This point deserves further investigation.

### Omeprazole’s Mechanism of Action in Nitrogen Use Efficiency

We evaluated the ability of OP to act as an ATPase inhibitor in maize. The inhibitory effect was only observed at concentrations 100- 1,000-fold higher than 1 µM dose that alleviated N starvation. Previous experiments in tomato have shown that concentrations in excess of 30 µM inhibit growth (Van Oosten et al., 2017a) and we have observed similar results in Arabidopsis (unpublished results). In animals, omeprazole and other benzimidazole PPIs interact with three cysteines in the beta loop of gastric Type IIC ATPases. Plants lack this class of ATPases (Axelsen and Palmgren, 1998; Hasegawa, 2013) and amino acid sequence analysis of mammalian Type IIC ATPases, in the region where OP binds, does not demonstrate any significant similarity with any regions in plant ATPases (unpublished results). While our results indicate that the concentration used to elicit enhanced salt tolerance (Van Oosten et al., 2017; Carillo et al., 2019d; Cirillo et al., 2019) and enhanced NUE does not affect total ATPase activity, it is clear that OP has an inhibitory effect at high concentrations. The phenotypes observed in this study and others at low OP concentrations are likely not due to OP having a direct effect on ATPase activity. The inhibitory effect of OP on ATPase activity in plants merits further study, since plants lack the Type IIC ATPases or similar proteins.

In maize, low doses appear to increase NUE. The enhanced ability to utilize limiting N conferred by OP treatment could be due to a combination of two major factors: uptake and reductive assimilation, in particular NO_3_^-^ reduction. We assayed root uptake in young root segments (cut roots) and observed no increased uptake on OP treatment (**Supplementary Figure 1**), even if the expression of key genes involved in nitrate uptake and plasma membrane ATPase, responsible for generating the proton motive force necessary for nitrate uptake, were upregulated (**Figure 6**). However, while OP treatment did not increase the rate of uptake, it did affect key steps in nitrate assimilation. OP treatment did increase assimilation through the reduction of nitrate to nitrite through a direct effect on NR. NR enzyme from OP treated plants was found to be in the constitutively active state, regardless of N status in the environment (**Figure 4**, **Table 2**). When OP was used in an *in vitro* enzyme assay using purified NR from *Arabidopsis thaliana,* higher concentrations of OP were able to increase the catalytic efficiency and the specificity for the substrate nitrate of the enzyme resulting in an increased V_max_ and decreased K_m_. This suggests that OP helps in maintaining adequate affinity of the enzyme toward its substrate as well as its catalytic rate (**Figure 5**). These two results indicate that OP likely directly and physically interacts with NR. This physical interaction that increases the affinity of the enzyme for its substrate may also interfere with inhibition of NR *in vivo*. NR undergoes a partial kinase-dependent inhibition, due to a phosphorylation, followed by an interaction with Mg ions and recruitment of 14:3:3 proteins, which decreases the enzyme activity and makes it more susceptible to proteolytic degradation (Kaiser et al., 1999). Nitrate in the cytosol is also able to protect the enzyme against proteolytic degradation (Campbell, 1999).

**Figure 9:**
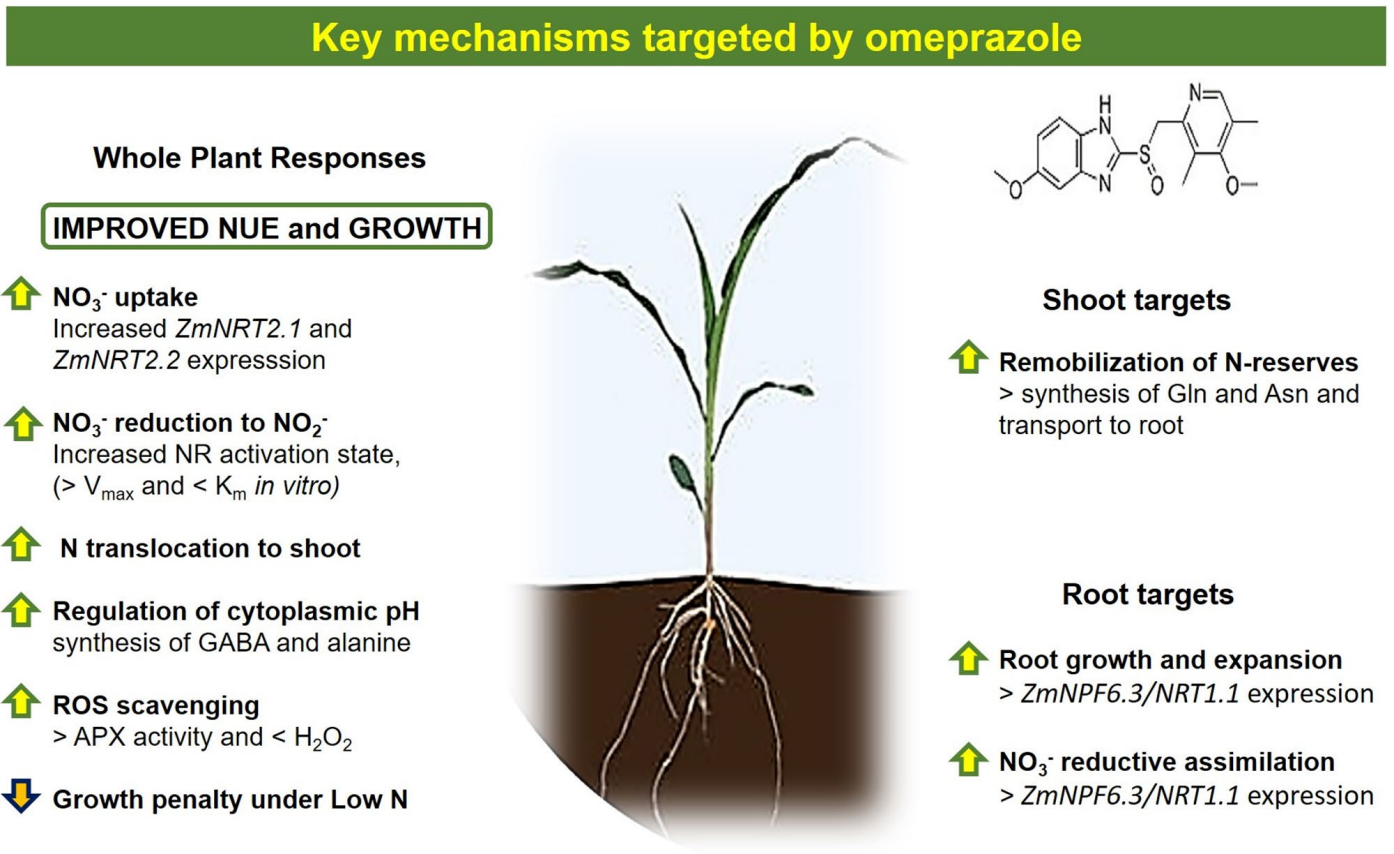
Summary of main key mechanisms targeted by omeprazole in corn.

Moreover, even if OP did not directly increase nitrate uptake in young root segments (cut roots), it could be responsible for a direct stimulation of root biomass, that is root surface and length by OP-induced endogenous phytohormones like auxin as suggested by Rouphael et al. (2018) or by OP-induced GABA (Van Oosten et al., 2017a). In support of this hypothesis, a strong increase of expression of *ZmNPF6.3/NRT1.1* in roots of OP treated plants was found. The phosphorylated form of NPF6.3 can be responsible for NPF6.3 dependent regulation of both nitrate and auxin transport and therefore for lateral root growth and expansion (Bouguyon et al., 2015). The increase of root dimension and biomass and therefore of available root surface could improve/maximize the efficiency of root, in terms of soil exploration and nutrient mobilization and acquisition, avoiding the loss of nitrate, which is highly soluble in water and prone to leaching, from the root zone (**Figure 9**). Indeed, a measurement of the hormonal balance within the plant upon OP application would be of great interest and could help to better understand the results obtained. Preliminary evidence suggests that OP interacts with plant hormones involved in both growth and stress responses and therefore indirectly with NUE (unpublished results).

### Implications of Omeprazole as a Regulator of NUE

Omeprazole treatment in maize plants alleviates the growth limitations imposed by low nitrogen in the environment (**Figures 1** and **2**). The changes in primary and secondary metabolism (**Figures 7** and **8**) are significant and the potential for enhancing NUE in field conditions needs to be determined. While benzimidazoles have been used in agriculture for decades as fungicides (Lucas et al., 2015), it is uncertain if public opinion and the current regulatory climate would accept a novel plant growth regulator like omeprazole and/or its derivatives. However, with the advances in gene editing, the targets of OP (**Figure 9**) make excellent candidates for gene editing with the aim of enhancing NUE in crop species. Our results show that it is possible to perturb the physiological process in the plant in such a way that uptake and assimilation can be enhanced through mechanisms present in the plant. Understanding how to regulate these processes is essential to enhancing NUE and subsequently developing sustainable crops with lower environmental impacts.

## Data Availability Statement

All datasets generated for this study are included in the article/**Supplementary Material**.

## Author Contributions

MO, ED’A, and PC contributed equally to this work. MO and PC contributed to the experimental design and wrote the first draft of article. MO, ED’A, AR, VC, PW, and PC performed the experiments. MO and PC analyzed the data. MO, YG, AM, and PC supervised and completed the writing. All authors have read and approved the article.

## Funding

Financial support by the Access to Research Infrastructures activity in the Horizon2020 Programme of the EU (EPPN2020 Grant Agreement 731013) is gratefully acknowledged. Authors also acknowledge the University of Campania “Luigi Vanvitelli” Programma VALERE: Vanvitelli per la Ricerca.

## Conflict of Interest

The authors declare that the research was conducted in the absence of any commercial or financial relationships that could be construed as a potential conflict of interest.
