# Supplementary material for: Omeprazole Treatment Enhances Nitrogen Use Efficiency Through Increased Nitrogen Uptake and Assimilation in Corn
Source: Front Plant Sci. 2019 Dec 4;10:1507. doi: 10.3389/fpls.2019.01507 (PMC6904362; doi:10.3389/fpls.2019.01507)
Supplement: Supplementary file 1 [file Table_1.docx]

**Supplementary Material**

Omeprazole treatment enhances NUE through increased nitrogen uptake and assimilation in corn

Supplementary Figure 1. Net nitrate uptake of detached maize roots. One gram of detached 3-week-old maize roots were incubated with 0, 100, and 500 µM NO_3_^-^ for 1 hour with and without, 0, 1, 10, 50, and 100 µM OP. The incubation solution was analyzed for nitrated content and values were normalized with 0 µM NO_3_^-^. Values indicate average ± SE (n=4) percent change over untreated controls.


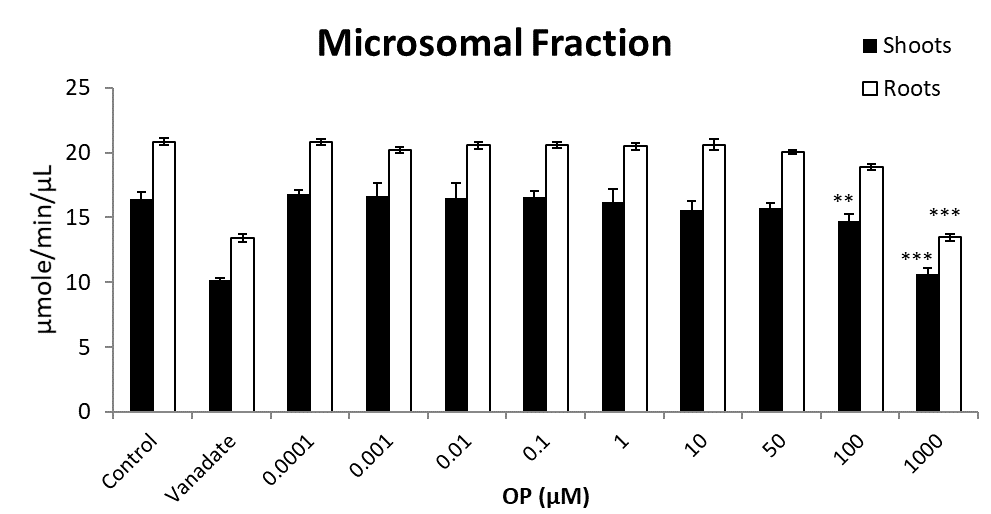


***

***

***


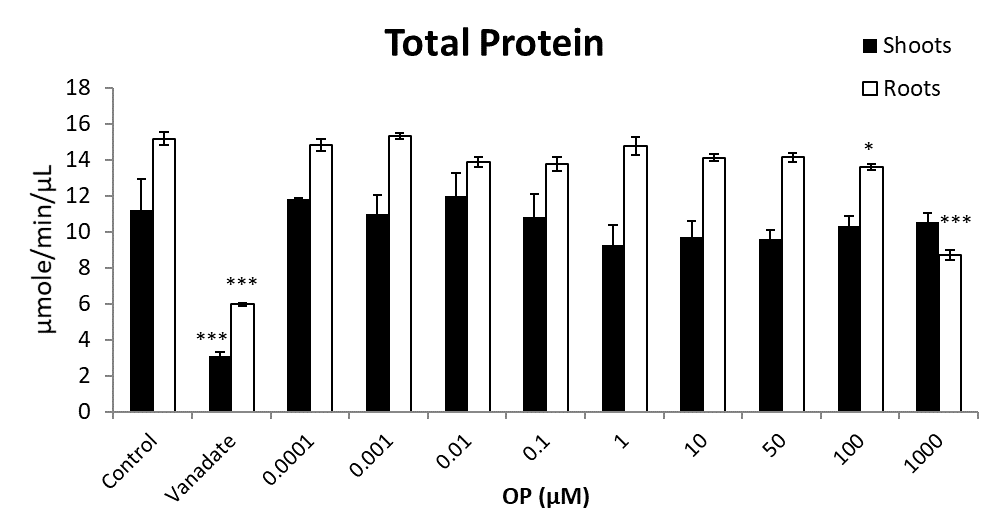


Supplementary Figure 2. ATPase activity of maize shoots and roots. Values indicate average ± SE (n=6). Single asterisks denote significant differences according to Student (P<0.05) between untreated controls and OP treated plants, double asterisks denote (P<0.01) between untreated controls and OP treated plants, triple asterisks denote (P<0.001) between untreated controls and OP treated plants.

Supplementary Table 1. Primers used in this study

| Primer Name | Primer sequence- 5’ to 3’ |
| --- | --- |
| ZmNRT2.1-F | ATCTTCGGGGTCATCCCCTTTGTCT |
| ZmNRT2.1-R | CAGCGTGCACGCCATGATCAT |
| ZmNRT1.5A-F | CGTATGTTGTTCTTGTCTTCTTG |
| ZmNRT1.5A-R | GTGCTATCGTCGTCAATGG |
| Zm-NRT2.2-F | CGACGAGAAGAGCAAGGGACT |
| Zm-NRT2.2-R | AGGTGAACATGGATGATGGAT |
| ZmNRT3.1A-F | GCATCCACGCCTCTCTCAAG |
| ZmNRT3.1A-R | TCAGCAACGACAGCCACTCAT |
| ZmNRt1.1/NPF6.3-F | CCGCCTATGAAATCGTCCTA |
| ZmNRT1.1/NPF6.3-R | GACCGTGTTGAGGTACGACCC |
| ZmNAR2.1-F | CTCGCCTTCTTCTTCGTCAT |
| ZmNAR2.1-R | ATCAGCAACGACAGCCACT |
| ZmNR-F | GGTGAAGATCAACGCGTGC |
| ZmNR-R | ATGTCTCGAGGTGCTTCT |
| ZmNiR-F | CTTCATGGGCTGCCTCAC |
| ZmNiR-R | GTAGACGTCGGCCAGGTG |
| ZmASNS4-F | CTCTACGACACGCGAGACAG |
| ZmASNS4-R | CGTTCAGCGCCTTCATCTCG |
| ZmMHA3-F | GAGAACAAGACCGCCTTCAC |
| ZmMHA3-R | AAGACGGGTACCCAACCATA |
| ZmMOLqRT_FOR2 | CTGTGTCCTCCGTGCTCCAT |
| ZmMOLqRT_REV2 | AGGACTCCCGCATCTCCATA |
